# Quantitative analysis of changes in the phosphoproteome of maize induced by the plant hormone salicylic acid

**DOI:** 10.1038/srep18155

**Published:** 2015-12-11

**Authors:** Liuji Wu, Xiuli Hu, Shunxi Wang, Lei Tian, Yanjie Pang, Zanping Han, Liancheng Wu, Yanhui Chen

**Affiliations:** 1 Henan Agricultural University and Synergetic Innovation Center of Henan Grain Crops, Zhengzhou 450002, China; 2 Key Laboratory of Physiological Ecology and Genetic Improvement of Food Crops in Henan Province, Zhengzhou 450002, China; 3 North China University of Water Resources and Electric Power, Zhengzhou 450046, China; 4 College of Agronomy, Henan University of Science and Technology, Luoyang 471003, China

## Abstract

Phytohormone salicylic acid (SA) plays an important role in regulating various physiological and biochemical processes. Our previous study identified several protein kinases responsive to SA, suggesting that phosphorylation events play an important role in the plant response to SA. In this study, we characterized the phosphoproteome of maize in response to SA using isotope tags for relative and absolute quantification (iTRAQ) technology and TiO2 enrichment method. Based on LC-MS/MS analysis, we found a total of 858 phosphoproteins among 1495 phosphopeptides. Among them, 291 phosphopeptides corresponding to 244 phosphoproteins were found to be significantly changed after SA treatment. The phosphoproteins identified are involved in a wide range of biological processes, which indicate that the response to SA encompasses a reformatting of major cellular processes. Furthermore, some of the phosphoproteins which were not previously known to be involved with SA were found to have significantly changed phosphorylation levels. Many of these changes are phosphorylation decreases, indicating that other currently unknown SA signaling pathways that result in decreased phosphorylation of downstream targets must be involved. Our study represents the first attempt at global phosphoproteome profiling in response to SA, and provides a better understanding of the molecular mechanisms regulated by SA.

The phytohormone salicylic acid (SA) plays an important role in regulating various physiological and biochemical processes, including plant defense, photosynthesis, cell expansion, respiration, thermotolerance, stomatal responses and nodulation^1^,^2^,^3^. Moreover, SA is an important signaling molecule for plant immune response and is necessary for the activation of SAR (systemic acquired resistance)^4^,^5^. However, the effect of SA on these processes may be indirect, because SA is heavily involved in crosstalk with other plant hormones^6^. These signaling networks and processes involve many protein to protein and protein to non-protein interactions^1^. Research on SA effects at the transcriptional and protein levels is abundant, but a more comprehensive picture of the change in phosphorylation induced by SA is needed.

Reversible phosphorylation is one of the most common post-translational protein modifications, and is critical to the control of multiple cellular functions, including the stress/defense responses^7^. It is estimated that one-third of eukaryotic proteins are phosphorylated^8^. A wide range of signaling networks and processes, such as protein kinase activation, cell cycle progress, cellular differentiation and transformation, development, and hormone responses are regulated by the state of protein phosphorylation^9^. Similarly, a variety of proteins has been reported to be phosphorylated in response to stimuli^9^. Hence, obtaining a complete systems-level analysis of molecular events requires direct measurements of proteins, including post-translational modifications that may affect their function^10^.

Our previous studies have identified several proteins that are responsive to SA^2^. In this work, based on isotope tags for relative and absolute quantification (iTRAQ) technology and TiO2 enrichment method combined with LC-MS/MS analysis, we monitored the response to SA in the phosphoproteome of maize leaves. Phosphoproteins with significantly changed phosphorylation levels were analysed to reveal their biological significance induced by SA. Our data provide new and valuable insights into the plant response induced by SA.

## Results and Discussion

### Phosphopeptide and phosphoprotein identification

**Figure 1:**
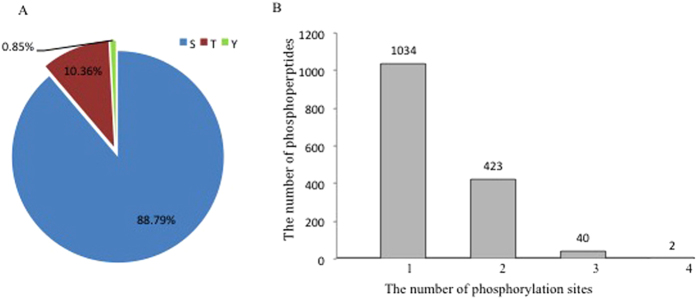
The distribution of phosphorylation sites. (**A**) Distribution of phosphorylation on serine, threonine, and tyrosine was assessed for all non-redundant localized phosphorylation sites. (**B**) Distribution of single- and multi-phosphorylated peptides showed that the majority of phosphopeptides have only one phosphorylation site.

About 1878 phosphopeptides were identified across the maize database (data not shown). Then, we removed the redundant and invalid peptides (PhosphoRS probabilities ≥75% and PhosphoRS scores ≥50). Thus, we identified 1495 unique phosphopeptides, collectively containing 2008 non-redundant phosphorylation sites (Original data for all the identified and quantified phosphopeptides were shown in Supporting Information Table S1). Among those phosphorylation sites, 1783 (88.79%) were found at serine, 208 (10.36%) at threonine and 17 (0.85%) at tyrosine residues (Fig. 1A). Among the 1495 unique phosphopeptides, 1034 were singly phosphorylated, 423 were twice phosphorylated, 40 were phosphorylated at three sites, and only 2 were found phosphorylated at four sites (Fig. 1B). The contribution of phospho-Ser (pS) was consistent with results from rice (89.5%)^11^ and the contribution of phosphor-Thr (pT) was similar to that found in *Arabidopsis* (9.9%)^11^. Moreover, the distribution of tyrosine phosphorylation in maize was 0.85%, which was similar to that in cotton (0.8%)^12^ and was in agreement with the previous report from maize^10^,^13^.

**Figure 2:**
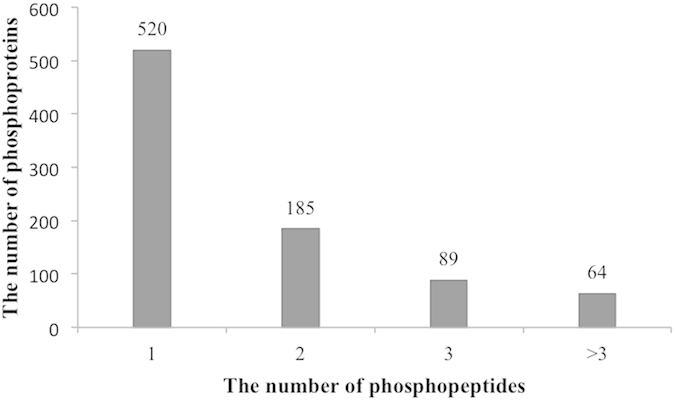
The distribution of phosphoproteins based on identification by single or multi phosphopeptide.

From the 1495 phosphopeptides, 858 phosphoproteins were identified. All the mass spectrometry data have been deposited to the ProteomeXchange Consortium via the PRIDE partner repository with the dataset identifier PXD002586. Passing through several steps in phosphopeptide identification, like protein digestion, phosphopeptide enrichment, fractionation and mass spectrometry analysis, it is possible that one or more phosphopeptides can be found in LC-MS analysis in one protein. In our study, among the 858 phosphoproteins, 520 were represented by a single phosphopeptide, 185 by two, 89 by three, and 64 by more than three phosphopeptides (Fig. 2).

### Quantitative analysis of phosphoproteins with phosphorylation levels significantly changed in response to SA

For quantitative analysis of phosphoproteins in response to SA, the change (treatment/control) of each phosphopeptide at 1, 4 and 9 h after SA treatment was calculated. An ANOVA with post hoc test was also performed which confirmed that there were significant differences among the three time points. Variance analysis results of these three periods were shown in supporting information Table S2. Of the analyzed samples, 291 phosphopeptides (244 phosphoproteins) showed a significant change (*P *<0.05, ratio ≥1.5, FDR <0.05) after SA treatment. Detailed information regarding phosphopeptides with statistically significant differences in maize after SA treatment was shown in Supporting Information Table S3. To graphically represent these *t*-test data, volcano plot –log_10_ (*P* value) *vs.* log_2_ (fold change) was constructed to graphically display the quantitative data (Supporting information Figure S2). The number of significantly changed phosphopeptides was 100, 150, and 160 after 1, 4, and 9 h, respectively. Some of the important phosphopeptides were shown in Table 1. In addition, the volcano plots indicate that some interesting peptides might be lost. Thus, it should be noted that this study cannot represent an exhaustive survey of all possible phosphorylation changes. A better approach would be to also accept lower abundance changes and confirm them in an independent experiment with higher number of biological replicates.

**Figure 3:**
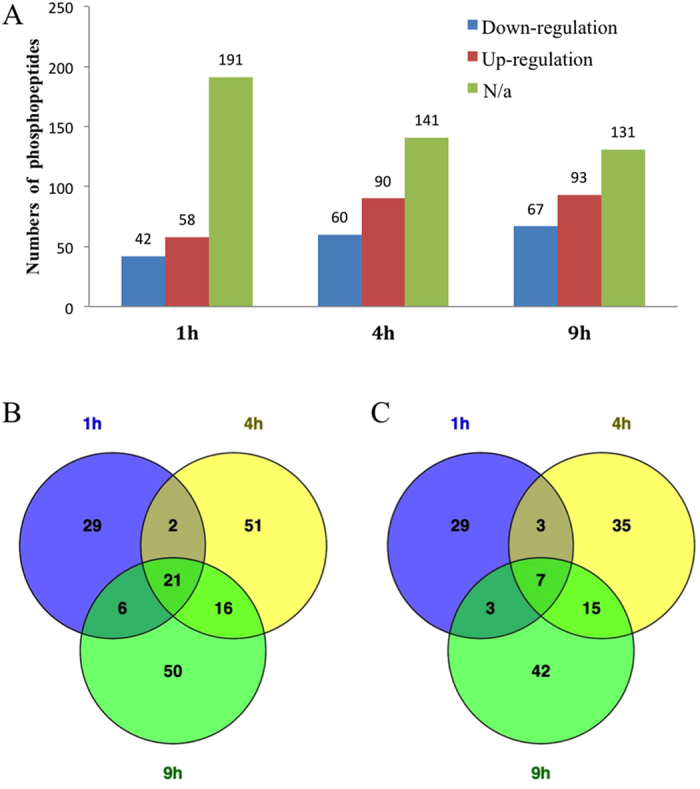
Analysis of the phosphopeptides whose levels changed after exposure to SA. (**A**) Numbers of phosphopeptides whose levels changed after exposure to SA for 1 h, 4 h and 9 h, respectively. N/a, not applicable, indicates that changes were not significant or were not detected in this group; (**B**) Venn diagram analyses of the up-regulated phosphopeptides in 1 h, 4 h and 9 h; (**C**) Venn diagram analyses of the down-regulated phosphopeptides in 1 h, 4 h and 9 h. Numbers in parentheses indicate the total number of phosphopeptides in 1 h, 4 h and 9 h, indicated by purple, yellow and green.

An increasing trend was found for both up- and down-regulated phosphopeptides, of which there were 42 and 58 after 1 h, 60 and 90 after 4 h, and 67 and 93 after 9 h, respectively (Fig. 3A). One reasonable explanation for this phenomenon is that following the SA treatment, an increasing number of phosphoproteins involved in signal transportation, but only a few phosphoproteins dephosphorylated during the analyzed time periods. In addition, Venn diagram analyses of the up- and down-regulated phosphopeptides were shown in Fig. 3B,C, respectively. There were 21 phosphopeptides whose levels were all up-regulated significantly at these three time points. On the other hand, there were seven phosphopeptides whose levels were all down-regulated significantly.

### Gene Ontology (GO) analysis

**Figure 4:**
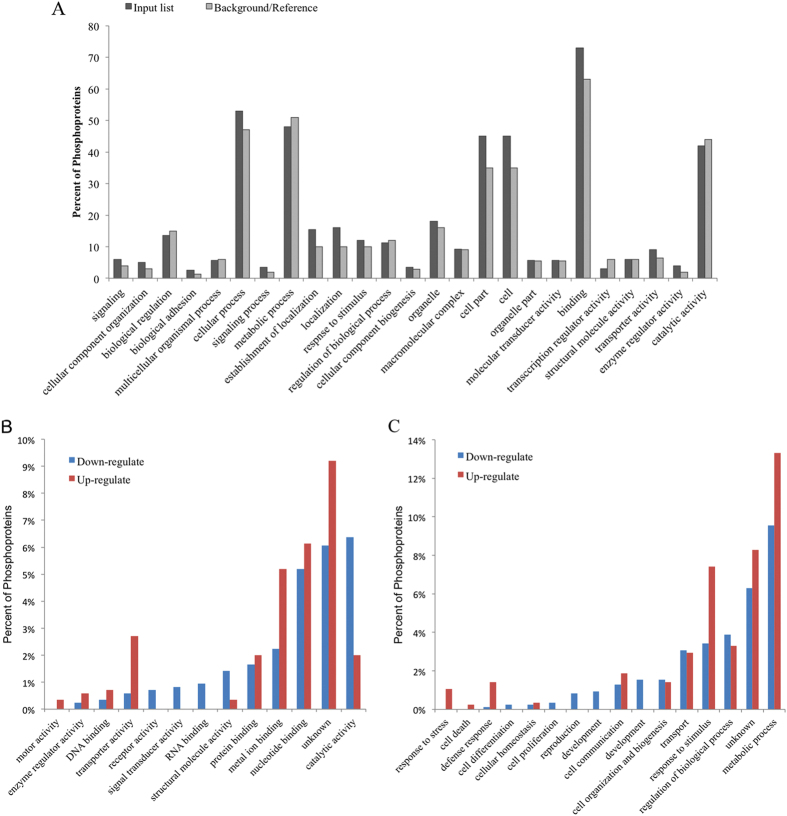
Phosphoproteins Gene Ontology (GO) distributions. (**A**) GO term distribution of the parent proteins of the identified peptides to the total genome; X-axis represents the GO annotation. Y-axis represents the phosphoproteins percentage of GO annotation. Three categories of GO annotation are biological process, cellular component and molecular function. The references/background represents all protein in AgriGO database. (**B**) Distribution of the up and down regulated phosphoproteins based on molecular function to all phosphoproteins identified. (**C**) Distribution of the up and down regulated phosphoproteins based on biological process to all phosphoproteins identified.

To reveal Gene Ontology (GO) distribution of phosphoproteins induced by SA in maize, functional enrichment analysis was used to assign biological relevance of these phosphoproteins using the Singular Enrichment Analysis (SEA) of AgriGO. The enriched GO annotations particularly concentrate on cellular component organization, cell part, cell, cellular process and binding (Fig. 4A, Table S4). By using the phosphoproteins whose phosphorylation levels were significantly regulated, GO analyses of up- and down- regulated phosphoproteins were performed. The 244 phosphoproteins can be classified into 13 functional categories based on their molecular function (Fig. 4B and Table S5). Compared to all phosphoproteins identified, the largest functional groups of the phosphoproteins with phosphorylation level up-regulated were unknown functional group (9.21%), nucleotide binding group (6.13%), metal ion binding group (5.19%), and a transporter activity group (2.71%). For the phosphoproteins with phosphorylation level down-regulated, the largest functional groups were catalytic activity (6.37%), unknown functional group (6.06%), nucleotide binding group (5.19%) and metal ion binding group (2.24%). The phosphoproteins could also be classified into 16 biological process groups (Fig. 4C and Table S5). The top four categories for the phosphoproteins with phosphorylation level up-regulated were metabolic processes (13.33%), unknown processes (8.28%), response to stimuli (7.43%) and regulation of biological processes (3.30%). For the phosphoproteins with phosphorylation level down-regulated, the top four categories were metabolic processes (9.55%), unknown processes (6.29%), regulation of biological processes (3.89%) and response to stimuli (3.42%).

**Figure 5:**
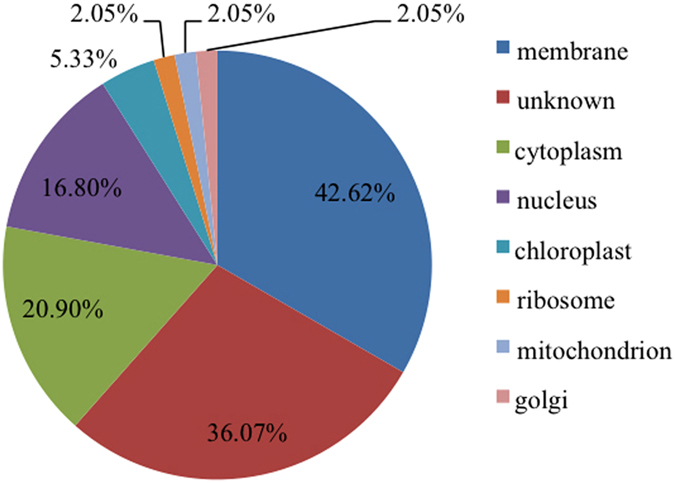
Phosphoprotein distribution based on cellular components. The percentage of differentially accumulated proteins was indicated.

Otherwise, the cellular components were membrane (104, 42.62%), unknown (88, 36.07%), cytoplasm (51, 20.90%), nucleus (41, 16.80%), chloroplast (13, 5.33%), golgi (5, 2.05%), ribosome (5, 2.05%), and mitochondrion (5, 2.05%) (Fig. 5). These results indicate that diverse cellular processes and cellular components are involved in the response to SA. However, note that sometimes one protein could be assigned to more than one GO category. Therefore, the sum of percentages in each category is over 100%. These results indicate that diverse biological processes and cellular components are involved in the response to SA.

### Phosphoproteins with catalytic activity

**Figure 6:**
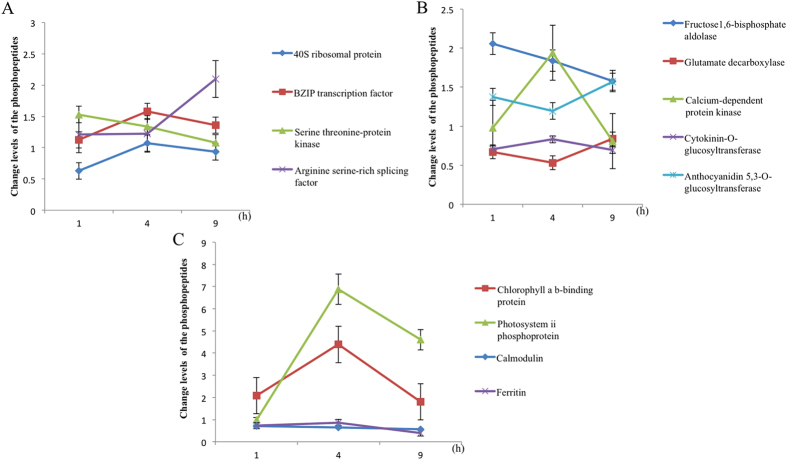
Change levels of the phosphopeptides significantly altered following SA treatment at 1 h, 4 h and 9 h. *n *= 3; error bar indicates SE. (**A**) phosphopeptides involved in catalytic processes; (**B**) phosphopeptides involved in nucleotide and protein binding; (**C**) phosphopeptides involved in metal ion bonding.

Many of the phosphoproteins that were significantly changed in phosphorylation levels in response to SA are involved in catalytic processes, including anthocyanidin, 5, 3-O-glucosyltransferase, cytokinin-O-glucosyltransferase, calcium-dependent protein kinase, glutamate decarboxylase, and fructose-bisphosphate aldolase. Traditionally, we think that anthocyanins modify flower and fruit color by the production of blue anthocyanins. However, in recent years, many links between anthocyanins and plant resistance have been found^14^. 3-deoxyanthocyanidin has been proved to be a phytoalexin important for resistance to fungi in a wide range of plants, such as apple, pear and *Sorghum bicolor*^15^,^16^. 5-O-glucosyltransferase (5-UGT) is a key enzyme in anthocyanin synthesis and is also related to plant resistance. In potato cells, over-expression of 5-O-glucosyltransferase can greatly increase the content of anthocyanins and sucrose, and confer significant resistance to bacterial infection^17^. In our study, after SA treatment, the phosphorylation level of anthocyanidin and 5, 3-O-glucosyltransferase showed a steady decline from 1 to 4 h, and a sharp increase from 4 to 9 h, reaching a significant level at 9 h after SA treatment (Fig. 6A). These results indicate that the phosphorylation or dephosphorylation of anthocyanidin and 5, 3-O-glucosyltransferase may play an important role in the late response to SA treatment, maybe downstream in the signal pathway of SA.

In contrast to anthocyanidin and 5, 3-O-glucosyltransferase, cytokinin-O-glucosyltransferase showed a steady increase from 1 to 4 h and a steady decline from 4 to 9 h, but the phosphorylation level of cytokinin-O-glucosyltransferase was lower than that in the control during the entire treatment time (Fig. 6A). This result is perhaps due to more storage forms of cytokinin-O-glucosides being converted to bioactive cytokinins after SA treatment. Cytokinins belong to a plant growth regulator group with important functions at all phases of plant development, from seed germination to senescence, and also to stress response^18^. Exogenous cytokinins have been reported to increase tolerance to mild stress, speed up recovery, reduce the negative effects of water deficit, improve the recovery of stomatal conductance and net photosynthesis after rehydration^19^,^20^. Moreover, the transcription of many stress-induced genes can be stimulated by cytokinins^21^. Cytokinin-O-glucosyltransferase plays an important role in cytokinin-O-glucoside synthesis. Cytokinin-O-glucosides represent cytokinin storage forms, as they can be converted to bioactive cytokinins by the action of β-glucosidases in plants to confront adverse environmental conditions^22^. Over-expressed cytokinin-O-glucosyltransferase can increase the ABA and auxin content in *Nicotiana tabacum*, and improve stress tolerance^22^. Over-expressed cis-cytokinin-O-glucosyltransferase in rice results in short-shoot phenotypes, delay of leaf senescence, and a decrease in crown root number^23^. The significant regulation of phosphorylation of cytokinin-O-glucosyltransferase in this study might indicate that it’s phosphorylation or dephosphorylation may also have a physiological impact on the growth and development of maize.

Calcium-dependent protein kinase regulates the downstream components in calcium signaling pathways, and is involved in crosstalk with other biotic and abiotic signaling pathways in plants^24^. Over-expression of an *Oryza sativa* calcium-dependent protein kinase, OsCPK12, promotes *Oryza sativa*’s tolerance to salt stress by reducing the accumulation of hydrogen peroxide (H_2_O_2_) in the leaves, but increases sensitivity to abscisic acid (ABA) and susceptibility to blast fungus. It showed that calcium-dependent protein kinase functions in multiple signaling pathways, positively regulating salt tolerance and negatively modulating blast resistance^25^. Recently, it is also reported that the phosphorylation level of calcium-dependent protein kinase changed significantly under drought stress in maize^26^. In this study, the change in phosphorylation level of calcium-dependent protein kinase is more intense than the two phosphoproteins discussed above, with a sharp increase from 1 to 4 h, and a sharp decline from 4 to 9 h (Fig. 6A). These results suggest that the phosphorylation of calcium-dependent protein kinase may play a positive role at the beginning of SA treatment, but as time passes, the signal pathway collapses.

Glutamate decarboxylase (GAD) catalyzes the conversion of glutamate to γ-aminobutyric acid (GABA). Glutamate and GABA are linked with the tricarboxylic acid (TCA) cycle and are essential to the synthesis of other amino acids in plants. Therefore, the GAD enzyme may have a central role in primary metabolism and significant suppression of its expression could be lethal^27^. Moreover, GAD is involved in feedback controls of Ca^2+^-permeable channels to adjust intracellular GABA levels and thereby modulate pollen tube growth. Findings suggest that GAD activity linked with Ca^2+^-permeable channels relays an extracellular GABA signal and integrates multiple signal pathways to modulate tobacco pollen tube growth^28^. The change in phosphorylation level of GAD was similar to that of anthocyanidin 5, 3-O-glucosyltransferase, but the phosphorylation level of GAD was lower than that in the control (Fig. 6A). This result shows that SA treatment can damage the primary metabolism of maize via the phosphorylation or dephosphorylation of GAD.

Fructose 1, 6-biphosphate aldolase (FBA) is a key enzyme in plants, which is involved not only in glycolysis and gluconeogenesis in the cytoplasm, but also in the calvin cycle in plastids^29^. Research has shown that the over-expression of aldolase in plants increases photosynthetic rate, growth and biomass yields^30^. Other studies have shown that FBA plays important roles in plant growth, stress responses and development^29^. In addition, it is recently reported that phosphorylation level of this enzyme can be significantly increased under drought and heat combination stress^26^. In our study, the phosphorylation level of fructose 1, 6-biphosphate aldolase declined from 1 to 9 h, but was above control levels during the entire period of sampling (Fig. 6A). This indicates that SA treatment can damage the primary metabolism of maize, and plants need more energy to repair the damage. Gluconeogenesis consumes plenty of energy thus up-regulates the phosphorylation level of the fructosebisphosphate aldolase which may inhibit gluconeogenesis for keeping energy in plants.

### Nucleotide and protein binding

This study identified numerous phosphoproteins involved in nucleotide and protein binding, like the arginine serine-rich splicing factor, serine threonine-protein kinase, bZIP transcription factor, and 40S ribosomal protein. Arginine serine-rich (SR) proteins constitute a conserved family of RNA-binding proteins with roles in both constitutive and alternative splicing^31^. The expression of SR protein under different environmental cues suggests they have an important role in plant defense and HR-like cell death^32^. It has been reported that the phosphorylation level of arginine SR splicing factor was significantly changed both under biotic and abiotic stresses^26^,^33^. In this study, the phosphorylation level of the arginine SR splicing factor was relatively stable from 1 to 4 h after SA treatment, but from 4 to 9 h, there was a sharp increase (Fig. 6B). These results suggest that transcription and translation may be major targets for regulatory phosphorylation during the response to SA in maize.

Threonine-protein kinase plays an important role in plant development and in the plant’s response to various unfavorable environmental conditions^34^. Over-expression of the threonine-protein kinase gene can enhance multistress tolerance in plants^35^. In wheat response to drought stress, phosphoproteome analysis reveals two threonine protein kinases^36^. Phosphorylation level changes of threonine protein kinases were also revealed in maize response to drought and heat combination stress^26^. Phosphorylattion level of serine threonine-protein kinase declined from 1 to 9 h after SA treatment (Fig. 6B). One reasonable explanation of this phenomenon was that serine threonine-protein kinase was upstream of the SA signal pathway.

The bZIP transcription factor is an important transcription factor regulating multiple biological processes including pathogen defense, responses to abiotic stresses, seed development and germination, senescence, and responses to salicylic, jasmonic, and abscisic acids in plants^37^. It has been shown that the phosphorylation of a bZIP transcription factor can act as positive regulators of ABA-responsive gene expression^38^. The expression of the bZIP transcription factor in rice can be rapidly induced by treating leaves with SA, suggesting that it plays a positive role in the SA-dependent signal transduction pathway for the defense of rice against pathogens^39^.

40S ribosomal protein is an important element of ribosomes in eukaryotes, and plays important roles in protein synthesis. It is also responsible for rapid adjustments in plant growth patterns under environmental changes^40^. Phosphorylation levels of two ribosomal proteins were differentially regulated in maize after virus infection, with 40S ribosomal protein up-regulated, and 60S ribosomal protein down-regulated^33^. In this study, the phosphorylation level of bZIP transcription factor showed a trend similar to that of the 40S ribosomal protein, increasing from 1 to 4 h and declining from 4 to 9 h after SA treatment. However, phosphorylation level of the bZIP transcription factor was up-regulated, whereas the 40S ribosomal protein was down-regulated, perhaps because the phosphorylation of bZIP transcription factor plays a positive role in the SA-dependent signal transduction pathway. SA can damage the protein synthesis system, so the phosphorylation of 40S ribosomal protein is down-regulated.

### Metal ion bonding

In this study, we also found some phosphoproteins with phosphorylation level changed significantly involved in metal ion bonding, like ferritin, photosystem II phosphoprotein, chlorophyll a/b-binding (LHCB) proteins, and calmodulin. Ferritins are molecules for iron storage and are present in most living things. In plants, ferritin is an essential iron homeostasis regulator and therefore plays a fundamental role in the control of iron induced by oxidative stress or by an excess of iron ions. Ferritin gene expression is modulated by various environmental factors, including the intensity of drought, cold, light and pathogenic attack^41^. It has also been reported that the phosphorylation level of ferritin was significantly changed under virus infection in maize, which indicated that the phosphorylation of ferredoxin might play a positive roles in maize for the resistance to pathogen infection^33^.

The primary reactions of plant and algal photosynthesis occur in the thylakoid membranes of chloroplasts. In the linear mode of electron transfer through the photosynthetic chain, photosystem II (PSII) and photosystem I (PSI) are connected in series through plastoquinone^42^. Phosphorylation of reaction-centre proteins affects their stability, possibly by slowing down their rate of degradation^43^. The light-harvesting chlorophyll a/b-binding (LHCB) proteins are the apoproteins of the light-harvesting complex of photosystem II. Down-regulation of any of the six LHCB genes results in abscisic acid (ABA)-insensitive phenotypes in seed germination and post-germination growth, demonstrating that LHCB proteins are positively involved in these developmental processes in response to ABA^44^. The activation of calmodulin (CaM) stimulates the DNA-binding activity of heat shock (HS) transcription factors, as well as the accumulation of HS proteins so as to confer thermotolerance. CaM can bind with target proteins to alter their function, acting as part of a calcium signal transduction pathway. In addition, the phosphorylation of CaM can lead to important physiological consequences for the cell as the diverse phosphocalmodulin species have differential actions as compared to nonphosphorylated CaM when acting on different CaM-dependent systems^45^.

The phosphorylation changes of the four phosphoproteins in this study were variable. Phosphorylation levels of ferritin and calmodulin were down-regulated, and remained relatively stable from 1 to 9 h after SA treatment (Fig. 6C). This suggests that the phosphorylation of both ferritin and calmodulin may play a negative role in the SA signal pathyway. But the phosphorylation level of photosystem II phosphoprotein and chlorophyll a b-binding protein were up-regulated following SA treatment, showing a sharp increase from 1 to 4 h and a sharp decline from 4 to 9 h after SA treatment (Fig. 6C). This phenomenon may occur because the plant’s response to SA is an energy dissipation process, and with the energy dissipation, the photosystem will collapse. Therefore, the phosphorylation level of photosystem II phosphoprotein and chlorophyll a b-binding protein declined during the late stages of the plant response to SA (Fig. 6C).

### Other phosphorylated proteins

We also found that some phosphoproteins were involved in other processes. For example, the auxin efflux carrier and aquaporin PIP2-5 are involved in transporter activity, and there are some phosphoproteins with unknown molecular function, like the small heat shock protein (sHSPs) 22. Division and growth of most types of plant cells require an external source of auxin. The auxin efflux carrier plays a negative role in controlling the level of auxin in plant cells, and thus can affect cell division^46^. Aquaporin PIP2-5 is a plasma membrane intrinsic protein. Aquaporins are ubiquitously presented in living organisms including plants. It has been provided evidence for abiotic stress induced quantitative changes in aquaporin phosphorylation and its link with sub-cellular localization^47^. Our previous study showed that the phosphorylation level of aquaporin was up-regulated by virus infection, indicating that aquaporin phosphorylation can also be induced by biotic stress^33^.

Heat shock proteins are classified based on their molecular weight (HSP100, 90, 70, and 60, and sHSPs. sHSPs act as molecular chaperones and can bind thermally denatured proteins at their surface to maintain a folding-competent state^48^. The phosphorylation of sHSPs has been demonstrated in maize mitochondria^49^. In addition, recent study showed that phosphorylation levels of seven HSPs, including five small HSPs (sHSPs) and two HSP70s, changed significantly under drought and heat^26^. These results indicate that the phosphorylation of sHSPs appears to be important for the regulation of sHSP function in plant responses to stresses. This also suggests that the SA signal pathway in maize is a very complex biological process.

### Comparison of the phosphoproteomic and proteomic changes

It will be interesting to compare the general proteomic results to this phosphoproteome. But it is a pity that we don’t have the proteomic results with the same materials and growth conditions in this study. So here we make a comparison of the phosphoproteomic and proteomic changes in response to sugarcane mosaic virus (SCMV) infection, which we have done separately before^33^,^50^. A total of seven proteins identified in the previous proteomic investigation were also detected with up- or down-phosphorylation in phosphoproteomic study. These proteins include phosphoenolpyruvate carboxykinase, serine/threonine-protein kinase, photosystem reaction center protein, eukaryotic translation initiation factor, chlorophyll a/b-binding protein, ATP synthase subunit alpha, and 60S ribosomal protein. This comparison of phosphoproteomic and proteomic changes in response to SCMV infection suggests that most proteins with quantity change are not phosphoproteins or have no change in phosphorylation level. On the other hand, most differentially regulated phosphoproteins have modification level change instead of protein quantity change.

The consistency between protein expression levels and phosphorylation levels suggests that these proteins or phosphoproteins may be initially regulated at the protein expression level. Discrepancy between protein expression and protein phosphorylation levels has also been observed in several previous studies^13^,^51^,^52^. By way of explanation, it has been suggested that the abundance of a protein integrates phosphorylation processing, which modulates the quantity, temporal expression, localization, and efficiency of the final product in the cell.

At last, one limitation of the present study should be noted. Although this study is meant to identify a number of potential protein targets of the phosphorylation/dephosphorylation machinery involved in the SA response, it cannot represent an exhaustive survey of all possible phosphorylation changes. The nature of an untargeted phosphoproteomics survey using a data dependent acquisition method on a mass spectrometer is inherently biased toward the identification of higher-abundance peptides in the sample. However, we believe that we have achieved an acceptable level of dynamic range, because we are able to identify high-abundance proteins, such as 60s ribosomal protein, as well as low-abundance transcription factors, such as bZIP. Furthermore, most of our reported phosphopeptide changes were identified in three biological samples and at more than one time point (Table 1 and Table S1). In addition, not all the phosphorylation changes in this study are biologically important. Promiscuous phosphorylation is a well-known phenomenon that occurs during *in vitro* assays since the concentration and location of reactants can be artificially high, thereby resulting in non-natural reactivity and phosphorylation patterns unreflective of true in plant chemistry^53^. Therefore, the forward or reverse genetics experiments are needed to be performed in the future to verify that these phosphorylation changes are directly involved in response to SA.

## Conclusion

Protein phosphorylation has been found to play an important role in multiple cellular functions including stress/defense responses^7^. Phosphoproteomic studies on the molecular basis of regulatory mechanisms in plants will enhance our understanding of fundamental and complex biological processes and provide information that can be exploited for potential agricultural applications^54^. Our previous study identified several protein kinases responsive to SA^2^, suggesting that phosphorylation events play an important role in the plant response to SA. In the current study, we further characterized the phosphoproteomes of maize in response to SA treatment and provided a global analysis of protein phosphorylation regulated by SA. As we used intact plants instead of cell suspensions, we have obtained insight into some general physiological changes occurring in plants that mount a response to exogenous SA.

**Figure 7:**
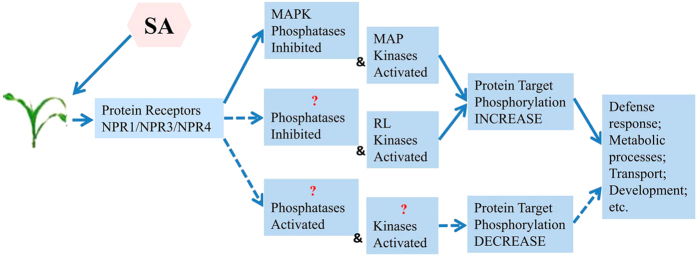
Mechanistic model for SA-induced changes in protein phosphorylation. Increased protein target phosphorylation is known to occur with activation of MAP kinases as well as from MAPK phosphatases inhibition or activation of RL kinases from an unknown phosphatases inhibition. Observed decreases in protein target phosphorylation could occur by the hypothetical activation of a phosphatase or inhibition of a kinase, which resulting from interaction with the SA receptor complex NPR1/NPR3/NPR4. Both phosphatase activation and kinase inhibition also may occur indirectly. NPR: Nonexpresser of pathogenesis-related genes; MAPK: Mitogen-activated protein kinases; RL kinases: Receptor-like protein kinases.

In summary, quantitative phosphoproteomic analysis of maize led to the identification of key phosphopeptides and phosphoproteins involved in the response to SA. We identified 858 phosphoproteins from 1495 phosphopeptides, among which 244 phosphoproteins were significantly differentially phosphorylated after SA treatment. While several of the phosphoproteins were well-known SA response phosphoproteins, many have not been reported previously. Our results also suggested that interaction with and inhibition of protein phosphatases may not be the only means of direct interaction of the SA receptor family with regulatory proteins, and other currently unknown SA signaling pathways that result in decreased phosphorylation of downstream targets must be involved (Fig. 7). The functions of the differentially accumulated phosphoproteins in SA signaling can be further tested using reverse genetic approaches. It will also be very interesting to identify the kinases that phosphorylate these phosphoproteins in response to SA.

## Methods

### Plant materials and hormonal treatments

Maize (*Zea mays* L.) seeds of inbred line B73 were grown in a greenhouse under a 16 h light/8 h dark cycle at 26 °C^55^. For hormonal treatments, the leaves of seedlings at the V4 development stage (when the fourth leaf has a visible “collar” at the base of the leaf) were sprayed with 10 ml of a solution containing 0.1% ethanol, 0.01% Tween-20 water solution and 200 μM SA (Sigma, USA). Each sample consisted of pooled leaves derived from six plants, with three biological replicates collected for each sample at 0 h, 1 h, 4 h, and 9 h after treatment. Samples were stored at −80 °C for further extraction of protein or RNA.

To gain a more comprehensive understanding of the maize cellular processes in response to SA, the iTRAQ technique was performed. We quantitatively compared phosphopeptides isolated from maize leaves from SA treatment and control samples. The workflow of the analysis is shown in Supporting Information Figure S1. Briefly, after extraction from SA and control treatments using a TCA-acetone procedure, protein was digested according to the FASP procedure. Then, the resulting peptide mixture was labeled using the iTRAQ reagent according to the manufacturer’s instructions. Finally, phosphorylated peptides were enriched by TiO_2_ beads, followed by lyophilization and MS analysis.

### Protein digestion

Total proteins were extracted from sample plants using a trichloroacetic acid/acetone procedure^33^. After quantification, protein digestion was performed according to the filter-aided sample preparation (FASP) procedure^57^. The FASP procedure is a method in which the sample is solubilized in 4% SDS, then retained and concentrated into microliter volumes in an ultrafiltration device. Notably, the presence of SDS efficiently inactivates detrimental enzymatic functions, such as protease and phosphatase activity^57^. The resulting peptide mixture was labeled using 4-plex iTRAQ reagent according to the manufacturer’s instructions (Applied Biosystems). Briefly, 200 μg of proteins from each sample were incorporated into 30 μl STD (4% SDS, 100 mM DTT and 150 mM Tris-HCl pH 8.0). The detergent, DTT and other low-molecular-weight components were removed using UA buffer (8 M urea and 150 mM tris-HCl, pH 8.0) by repeated ultrafiltration (Microcon units, 30 kDa). To block reduced cysteine residues, 100 μl iodoacetamide (0.05 M) in UA buffer was then added and the samples were incubated for 20 min in darkness. Filters were washed three times with 100 μl UA buffer, and then twice with 100 μl DS buffer (50 mM triethylammonium bicarbonate at pH 8.5). Finally, the protein suspensions were digested with 2 μg trypsin (Promega) in 40 μl DS buffer overnight at 37 °C with the resulting peptides collected as a filtrate.

### iTRAQ labeling

Peptide content was estimated by UV light spectral density at 280 nm using an extinction coefficient of 1.1 of 0.1% (g/l) solution that was calculated on the basis of the frequency of tryptophan and tyrosine in vertebrate proteins. For labeling, each iTRAQ reagent was dissolved in 70 μl ethanol and added to the respective peptide mixture. The samples, labeled as 114 (1 h), 115 (4 h), 116 (9 h), and 117 (0 h), were multiplexed and vacuum dried.

### Enrichment of phosphorylated peptides using TiO2 beads

Labeled peptides were mixed, vacuum-concentrated and resuspended in 500 μl loading buffer [2% glutamic acid, 65% acetonitrile (ACN), and 2% TFA]. TiO_2_ beads (GL Sciences, Japan) were then added and the mixture was agitated for 40 min. Centrifugation was carried out for 1 min at 5000 g, and the precipitated beads were set aside. The supernatant was mixed with another set of TiO_2_ beads, and centrifugation and bead collection was carried out a second time. Beads from the two centrifugation rounds were combined; they were washed three times with 50 μl of washing buffer I (30% ACN and 3% TFA) and three times with 50 μl of washing buffer II (80% ACN and 0.3% TFA) to remove remaining non-adsorbed material. The phosphopeptides were then eluted with 50 μl elution buffer (40% ACN and 15% NH_4_OH), followed by lyophilization and MS analysis.

### Mass spectrometry

Five microliters of the phosphopeptide solution were mixed with 15 μl of 0.1% (v/v) TFA; 10 μl of this mixture was analyzed for nanoLC-MS/MS using a Q Exactive mass spectrometer (Thermo Fisher Scientific) equipped with an Easy nLC HPLC (Proxeon Biosystems, now Thermo Fisher Scientific). The peptide mixture was loaded onto a C18-reversed phase column (15 cm long, 75 μm inner diameter, RP-C18 3 μm, packed in-house) in buffer A (0.1% formic acid) and separated with a linear gradient of buffer B (80% acetonitrile and 0.1% formic acid) at a flow rate of 250 nl min^−1^ controlled by IntelliFlow technology over 240 min. The peptides were eluted with a gradient of 0%–60% buffer B from 0 min to 200 min, 60% to 100% buffer B from 200 min to 216 min, and 100% buffer B from 216 min to 240 min.

For MS analysis, peptides were analyzed in positive ion mode. MS spectra were acquired using a data-dependent top ten method dynamically choosing the most abundant precursor ions from the survey scan (300–1800 m/z) for HCD fragmentation. The range of charge is from +2 to +6. Determination of the target value was based on predictive automatic gain control. The dynamic exclusion duration was 40 s. Survey scans were acquired at a resolution of 70,000 at m/z 200 and resolution for HCD spectra was set to 17,500 at m/z 200. The normalized collision energy was 27 eV and the under fill ratio, which specifies the minimum percentage of the target value likely to be reached at maximum fill time, was defined as 0.1%. The instrument was run with peptide recognition mode enabled.

### Data analysis

MS/MS spectra were searched using Mascot 2.2 (Matrix Science) embedded in Proteome Discoverer 1.4 against the UniProt_Poales, FASTA (426891 sequences) and decoy databases. For protein identification, the following options were used: peptide mass tolerance = 20 ppm, MS/MS tolerance = 0.1 Da, enzyme = trypsin, and missed cleavage = 2; carbamidomethyl (C), iTRAQ4/4plex (K) and iTRAQ4/4plex (N-term) were set as fixed modifications; oxidation (M), phosphorylation (S/T/Y), and false discovery rate (FDR) ≤0.01 were set as variable modifications. Proteome Discoverer 1.4 was used to extract the peak intensity within 20 ppm of each expected iTRAQ reporter ion from each fragmentation spectrum. Only spectra in which all the expected iTRAQ reporter ions were detected were used for quantification. The phosphopeptide ratios were normalized by dividing by the average value of all peptides identified. Phosphorylated peptides were analyzed using Proteome Discoverer 1.4 (Thermo Electron, San Jose, CA, USA) with the score threshold for peptide identification set at a 1% FDR and with PhosphoRS site probability cutoffs of 0.75. Phosphopeptide quantification was carried out in three biological replicates. Student’s *T* test was used to evaluate the statistical significance, and the false discovery rate (Benjamini-Hochberg) was calculated to correct for multiple comparisons. To state that a Phosphopeptide has a significant abundance changes, the following criteria have to be fulfilled: the abundance ratios has to be ≥1.5 and the *P-v*alue for student’s t test has to be less than 0.05.

### Bioinformatics

Molecular functions of identified phosphoproteins were classified according to their gene ontology (GO) annotations combined with their biological function. Subcellular locations of unique phosphoproteins identified in this study were determined from the UniProt database (http://www.uniprot.org) or predicted using the publicly available program, WolfPsort (http://wolfpsort.org).

## Additional Information

**How to cite this article**: Wu, L. *et al.* Quantitative analysis of changes in the phosphoproteome of maize induced by the plant hormone salicylic acid. *Sci. Rep.* **5**, 18155; doi: 10.1038/srep18155 (2015).

## Supplementary Material

Supplementary Information

Table S1

Table S3

Table S4

## Figures and Tables

**Table 1:** Phosphopeptides changed significantly in maize after SA treatment, as identified by LC-MS/MS.

Sequence^a^	Protein Group Accessions^b^	Gene ID	Description	phosphoRS Site Probabilities^c^	IonScore	114-117		115-117		116-117	
						Ratio^d^	p-value	Ratio	p-value	Ratio	p-value
**Response to stimulus**											
SAPTTPIKDGAASTFAAALSEEER	B7ZZR2	100279748	Phosphoenolpyruvate carboxykinase homolog2	S(1): 100.0; T(5): 97.2	52.86	0.48	1.75E-01				
SAPSTPKRSAPTTPIK	C0P3W9	541622	Phosphoenolpyruvate carboxykinase	T(4): 98.1; T(5): 100.0	22.6	1.21	6.50E-01	1.62	1.12E-01	1.68	1.28E-01
SAPSTPKRSAPTTPIK	C0P3W9	541622	Phosphoenolpyruvate carboxykinase	S(9): 99.5	22.6	1.21	6.50E-01	1.62	1.12E-01	1.68	1.28E-01
SAPSTPKRSAPTTPIK	C0P3W9	541622	Phosphoenolpyruvate carboxykinase	S(12): 95.3	22.6	1.21	6.50E-01	1.62	1.12E-01	1.68	1.28E-01
SAPSTPKRSAPTTPIK	C0P3W9	541622	Phosphoenolpyruvate carboxykinase	S(1): 99.5; T(5): 99.5; S(13): 100.0	22.6	1.21	6.50E-01	1.62	1.12E-01	1.68	1.28E-01
SAPSTPKRSAPTTPIK	C0P3W9	541622	Phosphoenolpyruvate carboxykinase	S(9): 100.0	22.6	1.21	6.50E-01	1.62	1.12E-01	1.68	1.28E-01
SAPSTPKRSAPTTPIK	C0P3W9	541622	Phosphoenolpyruvate carboxykinase	S(1): 99.7; S(4): 94.9; T(5): 94.9	22.6	1.21	6.50E-01	1.62	1.12E-01	1.68	1.28E-01
EAPTSSFYGGESPTPAPSLQGR	B7ZYS1	100285938	Tpa: phototropic-responsive nph3 family protein	S(12): 100.0	72.53	1.07	7.05E-01	0.61	3.02E-03	0.81	3.01E-03
DDGWGSSDDDTDAIDQDADPEADTTR	B6SQN8	100280985	Protein phosphatase inhibitor 2 containing protein	S(6): 100.0; S(7): 99.8	49.94	2.08	1.39E-01			1.7	5.90E-02
ARSEGEQEPVEPAPPVMASPLVAPGTPSGGASLK	B6SS20	100281086	Phototropin-1	S(3): 100.0; S(19): 100.0; T(26): 100.0	34.34	0.63	4.98E-01	0.57	4.32E-01	0.58	4.40E-01
ARSEGEQEPVEPAPPVMASPLVAPGTPSGGASLK	B6SS20	100281086	Phototropin-1	S(3): 100.0; T(26): 97.2	34.34	0.63	4.98E-01	0.57	4.32E-01	0.58	4.40E-01
LTSGQQISGEIPDASPDR	Q9LLI8	542564	Cellulose synthase-2	S(16): 98.5	28.71	0.66	4.62E-02	1.04	8.62E-01	1.24	1.08E-01
LTSGQQISGEIPDASPDR	Q9LLI8	542564	Cellulose synthase-2	S(8): 100.0; S(15): 100.0	28.71	0.66	4.62E-02	1.04	8.62E-01	1.24	1.08E-01
SGSGNIAELPDQGSLR	B4FY17	103626494	Phospholipase c	S(14): 100.0	58.79	0.66	3.91E-02	0.77	3.31E-01	0.78	2.42E-01
VGFPGASR	K7V096	103635738	E3 ubiquitin-protein ligase keg-like	S(7): 100.0	34.52	1.02	8.49E-01	1.53	4.52E-04	1.31	3.76E-03
SPGEVSGPESGQCEPEADADDNVR	C0HE46	100304252	Tho complex subunit 1-like	S(1): 100.0	32.36	1.16	6.42E-01	1.56	3.10E-01	0.82	5.94E-01
NNLTEGGAESDEEIR	F1DJV0	100192956	BZIP transcription factor	S(10): 100.0	60.21	1.13	1.08E-01	1.58	2.91E-04	1.36	2.21E-03
NEAGGIIGTRFESSDVK	K3ZVS1	101783042	Chlorophyll a b-binding protein	T(9): 100.0	28.83	2.09	1.34E-04	4.39	4.86E-06	1.8	9.48E-05
EAAEELSDGEK	K7TZ83	100501516	Sucrose-phosphate synthase family protein	S(3): 100.0	20.98	1.25	1.67E-01	1.43	4.63E-05	1.61	1.94E-04
EAAEELSDGEK	K7TZ83	100501516	Sucrose-phosphate synthase family protein	S(7): 100.0	20.98	1.25	1.67E-01	1.43	4.63E-05	1.61	1.94E-04
AQIATVR	K7UYG3	100383193	Uncharacterized protein	T(5): 100.0	38.37		3.80E-01	0.37	9.69E-02	0.79	4.79E-01
EGMESDDEIR	B6UEP1	100286123	Transcription factor HY5	S(5): 100.0	39.09	1.12	3.71E-01	0.65	1.99E-02	1.02	8.16E-01
EGMESDDEIR	B6UEP1	100286123	Transcription factor HY5	S(5): 100.0	39.09	1.12	3.71E-01	0.65	1.99E-02	1.02	8.16E-01
VSAGPGPENSGDDDPTVVEDSVAPQK	B4FLR0	100217168	GrpE protein homolog	S(10): 100.0	66	0.65	9.70E-02	0.79	2.78E-01	0.77	2.23E-01
APEPAEESDEEMGFSLFDD	B4FWI0	100273601	60s acidic ribosomal protein	S(8): 100.0	72.13	1.04	7.52E-01	0.52	8.79E-04	0.62	1.04E-03
AGSGGPDTPVFGDR	B6UBN4	100278637	J domain-containing protein required for chloroplast accumulation response 1-like isoform	S(3): 100.0	65.93	0.99	8.90E-01	1.33	2.51E-02	1.61	4.14E-02
AGSGGPDTPVFGDR	B6UBN4	100278637	J domain-containing protein required for chloroplast accumulation response 1-like isoform	S(3): 100.0	65.93	0.99	8.90E-01	1.33	2.51E-02	1.61	4.14E-02
IATADSQATSDDDEEQKQIEETTER	B6UCY8	100286002	Pre-mRNA-splicing factor prp45	S(10): 99.9	53.31	1.01	8.54E-01	1.26	2.44E-02	1.67	2.19E-02
GLDIETIQQSYTV	C4J9I5	100502231	Plasma membrane atpase 1-like	T(12): 100.0	58.9	1.25	4.80E-03	1.45	1.69E-02	1.61	2.77E-04
LELSAVPSSYYSGQLDPDR	C4JBW0	100502475	Glucose-6-phosphate 1-epimerase	S(12): 99.9	59.58	0.98	8.92E-01	0.52	1.33E-02	0.47	6.79E-03
SSLSESEKNDTPR	C5XUE0	8081975	Protein kiaa0664 homolog	S(4): 100.0	30.3	1.05	7.59E-01	0.69	8.80E-03	0.74	1.48E-02
SSLSESEKNDTPR	C5XUE0	8081975	Protein kiaa0664 homolog	S(4): 96.0; S(6): 96.0	30.3	1.05	7.59E-01	0.69	8.80E-03	0.74	1.48E-02
SLQSTAENNGIDLVK	K7TUQ9	103652775	Phosphatidate phosphatase lpin2-like	S(1): 99.9	30.3	0.82	4.10E-01	0.88	1.81E-01	0.27	1.10E-04
QPSEEPEEQVDLEGDDDGMDDDDAGYR	K7UBY5	100191663	Heterogeneous nuclear ribonucleoprotein r-like isoform	S(7): 99.9	61.42	1.52	2.91E-01			1.39	1.56E-02
QPSEEPEEQVDLEGDDDGMDDDDAGYR	K7UBY5	100191663	Heterogeneous nuclear ribonucleoprotein r-like isoform	S(3): 100.0	61.42	1.52	2.91E-01			1.39	1.56E-02
QPSEEPEEQVDLEGDDDGMDDDDAGYR	K7UBY5	100191663	Heterogeneous nuclear ribonucleoprotein r-like isoform	S(3): 100.0; S(7): 100.0	61.42	1.52	2.91E-01			1.39	1.56E-02
QPSEEPEEQVDLEGDDDGMDDDDAGYR	K7UBY5	100191663	Heterogeneous nuclear ribonucleoprotein r-like isoform	S(3): 100.0	61.42	1.52	2.91E-01			1.39	1.56E-02
VRPSTPVDMSPLPSPTK	K7USN0	100281191	2og-fe oxygenase family protein	S(10): 100.0; S(14): 99.9	26.13	0.76	1.18E-01	0.87	2.59E-01	0.55	1.28E-02
STSTPFMNTTDVGK	K7UTP6	542278	Nitrate reductase	S(6): 100.0	43.24	1.67	2.34E-01	1.59	3.79E-01	1.09	6.91E-01
STSTPFMNTTDVGK	K7UTP6	542278	Nitrate reductase	S(3): 94.5	43.24	1.67	2.34E-01	1.59	3.79E-01	1.09	6.91E-01
ALLLQSPSQVVAR	K7V0F9	103635158	Respiratory burst oxidase protein b	S(8): 100.0	45.89	0.57	2.95E-03	0.58	2.79E-01	0.87	3.39E-01
DSLDLSALGAAIPNSAELSAEDK	Q8L8G5	542588	Nucleosome/chromatin assembly factor group A	S(15): 100.0	70.35	1.55	2.07E-01	1.18	8.97E-02	1.35	1.41E-02
IEDSDDEEDLVPDAK	C5×6G2	8084990	Probable amp deaminase-like	S(4): 100.0	55.53	1.29	1.73E-01	1.32	4.69E-02	1.81	2.19E-03
IWQYGDVESDEDEQAPAAR	K3YPM5	101763417	Staphylococcal nuclease domain-containing protein 1-like isoform	S(9): 100.0	65.32	3.28	7.30E-03	2.11	5.94E-02	6.18	7.50E-07
EGGVESDEEIR	K7VQH0	103634871	Bzip transcription factor superfamily protein	S(6): 100.0	41.58	1.33	1.34E-01	1.48	1.05E-01	1.64	1.05E-04
VGSAADWSNQF	A2TJU6	101781720	Aluminum-induced protein-like protein	S(3): 100.0	51.98	1.56	1.50E-03	1.04	8.60E-01	1.32	1.54E-01
GKEEAGSADELDDEE	B4FAR1	100191940	Atpase 2	S(7): 100.0	44.45	0.99	9.73E-01	1.65	6.48E-03	1.61	4.62E-03
RSYTPDDINDR	B4FQ73	100856927	Sr repressor protein	S(2): 100.0; T(4): 100.0	51.97	1.03	6.66E-01	0.98	7.02E-01	0.67	3.43E-02
GLAASQEDLDR	Q5QJA2	100383635	Harpin binding protein 1	S(5): 100.0	23.51	1.14	3.05E-02	1.85	2.25E-04	1.5	7.43E-04
GLDIDTIQQNYTV	Q8L6A2	542052	Proton-exporting ATPase	S(5): 100.0	75.61	1.07	5.77E-01			1.52	7.72E-02
GLDIDTIQQNYTV	Q8L6A2	542052	Proton-exporting ATPase	T(12): 100.0	75.61	1.07	5.77E-01			1.52	7.72E-02
SGSSSSSSSEDDGMGGR	Q41299	542373	Dehydrin	S(3): 97.6; S(6): 99.9	108.71	2.21	2.91E-01	3.12	5.07E-02	2.44	2.29E-03
SGSSSSSSSEDDGMGGR	Q41299	542373	Dehydrin	S(3): 98.7	108.71	2.21	2.91E-01	3.12	5.07E-02	2.44	2.29E-03
ISSGEGPVSQGLSESGR	K7UHL6	103630022	Phosphate transporter pho1-3-like	S(15): 96.7	31.11	0.99	9.55E-01	0.64	8.46E-02	0.95	6.72E-01
GELALVPQSPDK	P29036	542553	Ferritin-1, chloroplastic	S(9): 100.0	46.1	0.73	3.07E-01	0.71	2.17E-01	0.47	5.49E-02
YEGAESEDDSVR	K7U9Y7	542602	Small heat shock protein 22	S(6): 100.0	52.19	2.12	1.07E-02	3.47	1.01E-04	16.57	1.46E-06
DIEASGPEAGEFSAK	B6T7B8	541890	Aquaporin PIP2.2	S(13): 100.0	60.71	0.97	7.24E-01	1.4	1.56E-02	1.55	1.34E-02
GQSALGSALGLISR	B6U6U2	100285573	Hexose transporter	S(3): 100.0; S(7): 100.0; S(13): 100.0	80.09	1.34	2.84E-01	2.3	2.08E-03	1.12	7.47E-01
GQSALGSALGLISR	B6U6U2	100285573	Hexose transporter	S(7): 100.0	80.09	1.34	2.84E-01	2.3	2.08E-03	1.12	7.47E-01
GQSALGSALGLISR	B6U6U2	100285573	Hexose transporter	S(7): 97.7; S(20): 100.0; S(24): 100.0	80.09	1.34	2.84E-01	2.3	2.08E-03	1.12	7.47E-01
GQSALGSALGLISR	B6U6U2	100285573	Hexose transporter	S(3): 100.0; S(13): 100.0	80.09	1.34	2.84E-01	2.3	2.08E-03	1.12	7.47E-01
AQEEDQSASANTSDSEPEPHDEL	B8A0J2	100279899	Growth regulator	S(7): 96.3	30.9	1.57	1.27E-01	1.63	1.77E-03	1.65	8.52E-04
QDIEASGPEAGEFSAK	Q9ATM7	541889	Aquaporin PIP2-3	S(14): 100.0	35.35	0.56	2.46E-02	0.99	8.58E-01	0.93	4.08E-01
LGSSASFSR	Q9XF58	542619	Aquaporin PIP2-5	S(6): 100.0	33.57	1.93	3.36E-05	1.62	2.24E-04	1.71	1.12E-03
**Metabolic process**											
SFDELSDDDDVYEDSD	B4FQK5	100272775	Eukaryotic peptide chain release factor subunit 1-1	S(6): 100.0	65.76		1.92E-01	0.47	1.16E-01	1.32	5.41E-01
TRSNISLDGDDDPYSGNQTER	C0HHJ0	542102	Set domain protein sdg111	S(6): 98.5	20.12	0.59	4.35E-01			0.99	9.83E-01
TNSRGSDIGLAK	C5WW43	8057104	Probable cellulose synthase a catalytic subunit 2	T(1): 97.6; S(6): 100.0	28.36	0.62	1.63E-02				
LAADNAGDTEASPR	B4FAA8	100192037	Transmembrane protein 115	S(12): 100.0	86.02	0.77	7.80E-02	0.67	1.01E-03	0.69	6.11E-06
VVSTQAPVQLGSLR	B4FBC2	100192102	Tpa: gdp-mannose -epimerase 1	S(12): 100.0	41.32	0.57	1.57E-02	0.88	8.34E-02	0.92	4.34E-01
NLQGHLGHGSE	B4FG90	100193773	Anthocyanidin 5,3-O-glucosyltransferase	S(10): 100.0	25.99	1.38	4.42E-02	1.19	7.24E-02	1.57	3.59E-03
LLESNINEHSSEEDIR	B4FNU7	100272474	BSD domain containing protein	S(10): 100.0; S(11): 100.0	30.99	0.85	3.66E-01	0.68	1.01E-01	0.79	1.42E-01
DTLNDVESGSAR	B4FTT0	100273231	Lipid phosphate phosphatase 3	S(10): 100.0	61.33	1.12	3.78E-01	1.61	1.08E-03	1.68	1.87E-01
DPHAVTSVSPK	B4FUW1	100273353	Hemk methyltransferase family member 2	S(9): 99.8	25.17	0.69	1.33E-02	0.9	4.30E-02	0.9	3.83E-02
VEDLWEVAEPQLSPSEK	B6SHG6	100280507	AKIN gamma	S(13): 100.0	51.38	1.53	3.26E-01			2.15	4.33E-02
SESLAK	B6T1H0	103633434	40S ribosomal protein S6	S(4): 100.0; S(6): 100.0	25.02	0.85	5.77E-01	0.71	1.33E-01	0.55	1.45E-02
SESLAK	B6T1H0	103633434	40S ribosomal protein S6	S(3): 100.0	25.02	0.85	5.77E-01	0.71	1.33E-01	0.55	1.45E-02
AATSSCNADSE	B6U0×3	100191999	Cytokinin-O-glucosyltransferase	S(10): 100.0	31.62	0.71	8.35E-03	0.83	1.10E-03	0.7	5.12E-04
IEDIDPANDSDASEEGDGDGDDLSVR	B6U1Z4	100285203	ATP binding protein	S(10): 100.0; S(13): 100.0	46.53	0.7	6.58E-01	0.77	7.23E-01	0.68	6.21E-01
DPWGGPLEISNADSATDDDR	B6U4I3	100285405	Endo-1,4-beta-glucanase Cel1	S(14): 99.9	56.71	0.75	1.37E-01	0.76	2.04E-01	0.6	1.41E-01
AQEVPLYSGSDR	B6U753	100285607	Calcium-dependent protein kinase	S(10): 97.9	24.33	0.98	9.48E-01	1.94	1.19E-01	0.81	4.32E-01
DNTQPCFPCSGSGAQVCR	B6U7L5	100278360	Protein disulfide-isomerase lqy1-like	S(10): 97.4	20.35	1.6	8.96E-02	2.1	5.28E-03	1.83	1.06E-02
DREEGEDLSDGGAAEK	B6U7Y7	100285671	USP39 protein	S(9): 100.0	41.87	0.75	1.57E-02	0.65	1.03E-02	1.15	1.24E-01
AEDEEHSSDFEPEENGEGADDEEIDEEDDDGEDSVK	B6U982	100283317	Immediate-early protein RSP40	S(7): 100.0; S(8): 100.0	62.43	2.9	1.54E-02	2.25	5.95E-02	2.66	2.47E-02
VFSQLSGGEGR	C0HEK2	100383535	Probable receptor-like protein kinase at5g24010-like	S(6): 100.0	30.89	1.01	9.01E-01	0.95	7.73E-01	1.65	2.48E-03
NEAGGIIGTRFESSEVK	C0HF02	542478	Photosystem ii subunit29	T(9): 100.0	33.67	1.7	2.69E-02	2.59	3.78E-04	4.01	6.93E-04
NEAGGIIGTRFESSEVK	C0HF02	542478	Photosystem ii subunit29	T(9): 100.0	33.67	1.7	2.69E-02	2.59	3.78E-04	4.01	6.93E-04
GGPDGSSAHQQLAVPENLDATMR	C0HIM6	100381755	Integrin-linked protein kinase family protein	S(6): 100.0; S(7): 100.0	44.83	1.12	4.46E-01	1.58	6.47E-03	1.72	2.72E-03
ASSPQTDAEQGGGSLK	C0P4D8	100286124	Uncharacterized protein	S(3): 100.0	33.2	1.16	1.89E-01	0.95	2.79E-01	0.63	6.19E-04
EVSSDDDFVMPTAQK	C0P8K4	100276864	Dna ligase 1-like	S(3): 100.0; S(4): 100.0	32.66	0.69	9.91E-02	0.54	1.33E-02	0.52	6.10E-03
VPSGDLAALAAAESSER	C0PCC7	100191549	Glutamate decarboxylase	S(3): 100.0	73.3	0.67	1.38E-02	0.53	9.97E-04	0.84	4.66E-02
GLVPLAGSNNESWCQGLDGLASR	C0PD30	100273196	Fructose1,6-bisphosphate aldolase	S(12): 100.0	76.71	2.06	9.29E-02	1.84	2.41E-01	1.58	2.51E-01
SYTNLLDLAEGNFAALGPAAGAGGGGR	C0PDN0	103636651	Tpa: trehalose phosphatase synthase family protein	S(1): 97.7	79.22	0.9	3.11E-01	1.37	3.02E-01	1.57	3.35E-01
STGSTEEFFVR	C0PF64	101027210	Protein phosphatase regulatory subunit-like protein	S(1): 100.0; S(4): 97.4	33.35	0.73	1.04E-02	0.73	7.30E-03	0.56	3.46E-04
MQGLTGTGSPTVNHIER	C4J1N5	100217144	Endothelin-converting enzyme 2-like	S(9): 100.0	40.57	1.16	4.39E-01	1.69	1.13E-02	1.29	1.40E-01
AASSATAPAPADGSVSCGSPR	C4J6F5	100272884	Lymphoid organ expressed yellow head virus receptor protein	S(19): 97.0	28.77	1.33	1.84E-01	1.78	1.84E-01	2.83	1.91E-03
NFDTQESDSEEPSSIGDGDLVK	C4JAH4	100216636	Uncharacterized protein	S(7): 97.2	62.04	0.88	6.83E-01	1.57	5.53E-02	1.15	5.76E-01
QVADSAEGDEPSL	C4JBL2	100285980	Leucine-rich repeat receptor-like protein kinase family protein	S(5): 100.0	85.12	0.98	9.12E-01	0.67	3.85E-03	0.61	1.63E-03
QMSINSVPK	C5WR61	8057236	Serine/threonine-protein phosphatase	S(3): 100.0	29.62	1.04	7.14E-01	1.51	4.26E-02		
SELVSPASSPR	C5WRC6	8055610	Uncharacterized protein	S(5): 100.0; S(9): 97.9	43.91	1.22	6.10E-03	1.22	9.69E-02	2.23	4.58E-02
LLNADIQIGSPR	C5WZ61	8084177	Cytosolic purine 5-nucleotidase	S(10): 100.0	54.73	2.08	1.25E-02	1.51	1.09E-02	1.75	3.27E-03
GGIDSPSWR	C5×682	8063305	Pantothenate kinase 2-like	S(5): 100.0	27.15	1.12	2.20E-01	1.55	1.11E-02	1.11	4.96E-01
VGGGDGGDGGAAGDSADDGDAK	C5XCA3	8083903	Prp18 domain containing protein	S(15): 100.0	27.71	0.64	9.49E-02	0.87	2.77E-01	0.77	5.49E-02
SNNLSFK	C5XIB3	8059184	Type i inositol- -trisphosphate 5-phosphatase 1-like	S(5): 100.0	27.71	1.55	2.07E-04	1.49	5.25E-02	1.89	2.21E-02
TIGVSGLGEGSPK	C5YTD4	8065266	Tpa: protein kinase superfamily protein isoform 1	S(11): 100.0	36.96	0.87	4.93E-01	1.52	7.15E-02	1.69	2.15E-02
VEEKEESDEDMGFSLFD	C5YYT5	8072508	60s acidic ribosomal protein p2b	S(7): 100.0	27.28	0.92	6.83E-01	1.48	6.27E-02	1.69	7.72E-03
LAVAAPAAVQST	E3UMH7	100101542	Putative growth-regulating factor 1	S(11): 97.4	36.05	0.85	2.66E-01	0.7	1.39E-01	1.39	1.85E-01
GGFADEGSATTESDIEETLK	E9NQE1	542372	Phosphoenolpyruvate carboxylase	S(3): 100.0	83.85	1.11	5.10E-01	0.44	2.20E-04	0.53	2.08E-04
GGFADEGSATTESDIEETLK	E9NQE1	542372	Phosphoenolpyruvate carboxylase	S(13): 97.6	83.85	1.11	5.10E-01	0.44	2.20E-04	0.53	2.08E-04
DLNVVSPR	K3YP69	103626570	Microtubule-associated protein futsch-like isoform	S(6): 100.0	33.14	1.45	8.52E-02	1.54	4.36E-02	1.41	1.70E-01
GRSFGSSSLPR	K3ZPZ9	103632253	Uncharacterized protein	S(7): 93.9; S(8): 99.7	28.33	1.5	4.85E-02	1.34	4.67E-02	1.14	3.70E-01
TVQFVDWCPTGFK	K3ZT92	101753745	Tubulin alpha-3 chain-like	T(10): 100.0	52.8	0.58	8.41E-02	0.7	1.19E-01	0.78	2.13E-01
LSSDVGQLNVNK	K7U6F2	103642014	Putative translation elongation factor Tu family protein	S(3): 98.4	36.82	1.22	5.50E-01	1.77	4.40E-04	1.4	3.79E-02
ASSQASLADPDDFDLTR	K7U832	100281530	Neutral alkaline invertase	S(3): 99.8; S(7): 99.8	35.48	1.14	5.87E-01	1.6	6.16E-03	1.38	5.76E-03
ASSQASLADPDDFDLTR	K7U832	100281530	Neutral alkaline invertase	S(3): 99.9; S(6): 100.0	35.48	1.14	5.87E-01	1.6	6.16E-03	1.38	5.76E-03
NDPNYDSGEEPYELVEAPVSTPLEDYK	K7U9W6	100501384	Programmed cell death protein 4-like	S(7): 100.0	48.25	0.84	7.17E-01	0.55	3.73E-01	0.71	5.41E-01
VMSVASPASPTSPSPPAPPR	K7V0H0	103654185	Putative trehalose phosphatase/synthase family protein	T(11): 96.3; S(12): 96.5	29.02	0.46	1.27E-02	0.87	6.21E-01	0.59	3.17E-02
ESDLEAVGEPMSPAGR	K7V7L2	103651350	O-acyltransferase wsd1-like	S(12): 100.0	34.62	0.61	2.14E-01	1.07	6.81E-01		
AIAPAVGQSSGMAPVANNNR	K7VE03	103635432	Casein kinase family protein	S(9): 97.5	32.37	0.69	5.62E-01	1.28	5.08E-01	0.61	4.60E-01
RPSYSLSQNQNQAPPAAR	K7W2Z7	100192644	Protein kinase superfamily protein	S(7): 100.0	56.96	0.72	3.64E-02	0.77	1.86E-02	0.66	1.45E-02
SSSPFAASPPSPLR	K7W4F1	103636629	Putative HCNGP-related transcriptional regulator family protein	S(1): 94.1; S(11): 100.0	37.6	1.61	2.26E-01	1.25	3.72E-01	1.39	2.33E-01
SPLQNSPTFNR	K7W787	100273408	Spastin protein orthologue	T(8): 100.0	29.66	1.32	3.40E-01	1.78	4.16E-02	1.39	2.01E-01
SKLSAAAK	O04014	542529	40S ribosomal protein	S(4): 100.0	24.89	0.63	1.69E-02	1.07	8.42E-01	0.93	6.97E-01
GAGGGGGGGDPRSPTK	P31927	542711	Sucrose-phosphate synthase	S(11): 100.0	49.26	1.29	3.61E-01	1.31	2.65E-03	1.51	3.86E-02
GAGGGGGGGDPRSPTK	P31927	542711	Sucrose-phosphate synthase	S(3): 100.0	49.26	1.29	3.61E-01	1.31	2.65E-03	1.51	3.86E-02
GAGGGGGGGDPRSPTK	P31927	542711	Sucrose-phosphate synthase	S(11): 99.9	49.26	1.29	3.61E-01	1.31	2.65E-03	1.51	3.86E-02
GAGGGGGGGDPRSPTK	P31927	542711	Sucrose-phosphate synthase	S(3): 100.0; T(6): 100.0	49.26	1.29	3.61E-01	1.31	2.65E-03	1.51	3.86E-02
GAGGGGGGGDPRSPTK	P31927	542711	Sucrose-phosphate synthase	S(13): 100.0	49.26	1.29	3.61E-01	1.31	2.65E-03	1.51	3.86E-02
LTSGFQQFK	Q41729	542302	Carbonic anhydrase	S(3): 100.0	33.73	1.22	2.33E-01	1.92	1.37E-04	1.82	1.74E-04
VPIEAEMSEDTADDDISSR	K7VN24	100281651	Adhesion regulating molecule conserved region family protein	T(11): 100.0	49.85	1.54	3.06E-01	2.51	2.50E-02	1.84	2.06E-02
VPIEAEMSEDTADDDISSR	K7VN24	100281651	Adhesion regulating molecule conserved region family protein	S(8): 100.0; T(11): 100.0	49.85	1.54	3.06E-01	2.51	2.50E-02	1.84	2.06E-02
VRSPSPPLKK	M1H548	103650711	Arginine/serine-rich splicing factor	S(3): 100.0; S(5): 100.0	20.48	1.39	1.94E-01	1.51	3.56E-01	1.14	7.50E-01
LNQLNSSGGADDDDEDDE	B6THG9	103650892	60S ribosomal protein L5-1	S(6): 98.1	53.57	0.55	1.11E-01	0.58	1.54E-01	0.67	2.08E-01
**Regulation of biological process**											
NENPGLENLNHTPLSDAAAQNLSPR	K7V9×5	100217186	Uncharacterized protein	S(23): 100.0	49.52	0.9	5.48E-01	0.62	2.05E-02	0.67	3.26E-02
EKGDSDEEEDDYDK	C0PA70	100283725	Nuclear cap-binding protein subunit 2	S(5): 100.0	32.44	0.79	1.54E-02	0.64	2.69E-03	0.59	5.45E-04
ALAAGDSNASSSTYDMEDEESDDETTEEGGPVEEASSTGSDK	C0P9K3	100284226	Inner membrane protein albino3	S(11): 97.2	104.62	1.58	1.23E-01	1.21	3.57E-01	1.24	2.45E-01
VDQATPTEHGEEAGVK	C0P3W2	100282270	Kh domain containing protein	T(5): 96.9	20.55	0.86	1.82E-01	0.64	1.36E-03	0.7	8.00E-05
SSVIEGTQPEDDSDKEDVEAK	C5XU69	8064256	Pre-mrna-splicing factor atp-dependent rna helicase dhx16-like	S(13): 100.0	36.52	0.68	3.00E-03	1.1	5.89E-01	1.07	7.89E-01
SHQQGSGHGDDGDQESR	B4FNB7	100272384	Latex-abundant protein	S(6): 99.9	24.56	1.13	1.48E-01	1.72	4.37E-04	1.8	6.12E-05
SGGDVSEDSEDEKPLAAR	C6KE75	100191474	DNA topoisomerase I	S(6): 100.0; S(9): 100.0	54.32	0.97	5.88E-01	0.96	6.91E-01	1.06	2.95E-02
TIKESMDELNSQR	B4FVB8	100281683	Serine threonine-protein kinase	S(5): 100.0	29.1	1.53	1.01E-02	1.34	6.25E-03	1.08	8.13E-02
PDSDNDSGGPSNAGGELSSPR	B6STN2	100281247	Nuclear transcription factor Y subunit B-3	S(3): 99.9	57.63	2.55	2.67E-02	4.4	1.05E-04	0.8	3.78E-01
GGASSSGAAAQSPSSAPNKEPPQLSPADR	B6SXN6	100281671	Homeodomain protein JUBEL1	S(5): 95.5; S(12): 99.9	43.8	0.93	6.15E-01	0.68	5.79E-02	0.78	1.11E-01
VDFDEFGSMDATK	B6TPK0	100284050	CAK1AT	S(8): 100.0	20.65	0.92	7.32E-01	1.53	3.11E-04	1.34	4.89E-03
QIAGGDDSMDEQEDETQENVWGR	C0HIQ2	100381768	Something about silencing protein 10-like isoform x4	S(8): 100.0	24.74	1.14	4.79E-01	0.86	2.97E-01	0.67	4.36E-02
AQLPSPSPSPR	C5XP18	8057759	Tbc1 domain family member 8b-like	S(5): 100.0	27	0.67	1.39E-01	1.02	8.30E-01	0.96	5.32E-02
ATQTVEDSSRPK	K3ZC29	845205	Photosystem ii phosphoprotein	T(4): 100.0	46.85		1.14E-01	6.89	5.46E-05	4.61	5.91E-04
ATQTVEDSSRPK	K3ZC29	845205	Photosystem ii phosphoprotein	T(2): 100.0; T(4): 100.0	46.85		1.14E-01	6.89	5.46E-05	4.61	5.91E-04
ESEMDEHSGFSDGDGTGK	K7UKZ7	103630545	Transcription elongation factor spt6-like	S(11): 100.0	36.69	0.77	1.53E-01	1.02	8.03E-01	0.55	1.15E-02
DTDSEEELK	K7VGX4	100192601	Calmodulin	S(4): 100.0	42.15	0.71	2.03E-03	0.64	4.86E-05	0.57	2.85E-05
ATQTVEDSSRPKPK	P24993	845205	Photosystem II reaction center protein H	T(2): 100.0; T(4): 100.0	20.17	1.05	6.10E-01	1.5	1.47E-02	1.35	3.58E-03
ATQTVEDSSRPKPK	P24993	845205	Photosystem II reaction center protein H	T(4): 99.9	20.17	1.05	6.10E-01	1.5	1.47E-02	1.35	3.58E-03
DYNSDDEDEAGDDR	B4FYQ9	100280074	Uncharacterized protein	S(4): 98.2	31.79	0.99	9.34E-01	0.83	2.30E-01	0.65	5.19E-02
LQLGSDDEDGEGPYGSDADEGFEAGEGDNEELAIAR	B6SSK6	100191761	Nucleolar RNA helicase 2	S(7): 100.0	104.58	0.57	4.85E-01				
LQLGSDDEDGEGPYGSDADEGFEAGEGDNEELAIAR	B6SSK6	100191761	Nucleolar RNA helicase 2	S(16): 96.9	104.58	0.57	4.85E-01				
GRPAFGGPGFSPR	C0PDQ9	100383162	Flowering time control protein fca-like isoform x2	S(12): 100.0	38.56	1.36	1.07E-01	1.07	6.75E-01	1.73	5.62E-03
GRPAFGGPGFSPR	C0PDQ9	100383162	Flowering time control protein fca-like isoform x2	S(11): 100.0	38.56	1.36	1.07E-01	1.07	6.75E-01	1.73	5.62E-03
SGRESDSDADDIEHIEK	C5WPJ1	8054149	Inositol hexakisphosphate and diphosphoinositol-pentakisphosphate kinase 2-like	S(7): 100.0	32.87	1.21	3.37E-01	1.04	6.62E-01	0.53	1.11E-01
YGVDSFDPNR	C5YHC9	8069634	Tetraspanin-20-like isoform	S(5): 100.0	42.07	1.4	4.22E-01	1.65	4.23E-03	1.25	2.14E-01
VLSHSNESSNGASPEISPR	K3ZPW7	103627850	Tetratricopeptide repeat -containing protein	S(13): 97.8	28.33	0.81	5.87E-01	1.51	4.77E-02	1.13	5.44E-01
DVSNASELATEMQYER	K7TFK8	100384278	Uncharacterized protein	S(3): 100.0; S(6): 100.0	43.57	0.79	2.35E-01	1.13	1.67E-01	1.55	7.36E-03
GEPNISYICSR	K7U143	103641344	Putative glycogen synthase kinase family protein	Y(7): 98.5	40.03	1.14	1.49E-01	0.48	1.83E-03	0.74	1.88E-02
AASRDDGDFDDEEPR	K7U3F5	103641640	Low quality protein: exocyst complex component 2-like	S(3): 100.0	39.12	1.13	5.69E-01	0.86	1.14E-02	0.7	1.05E-03
ELAGAGPSLPSPTSDAR	K7UD94	103628994	Serine threonine protein phosphatase 2a 57 kda regulatory subunit b gamma isoform-like	S(11): 93.1	34.84	1.05	6.91E-01	1.15	3.20E-02	1.82	4.21E-05
VEQPFEEDEMDLDSEDEDEELNVPVVK	K7UM03	542355	Histone deacetylase hdt1	S(14): 100.0	68.93	0.82	3.07E-01	0.81	5.03E-01	0.66	2.05E-01
VEQPFEEDEMDLDSEDEDEELNVPVVK	K7UM03	542355	Histone deacetylase hdt1	S(14): 100.0	68.93	0.82	3.07E-01	0.81	5.03E-01	0.66	2.05E-01
SKDDSSSLASSISGSPR	K7UN30	103629018	Protein chloroplastic-like	S(9): 100.0	24.43	1.65	6.49E-03	1.41	2.54E-02	1.53	8.81E-03
SKDDSSSLASSISGSPR	K7UN30	103629018	Protein chloroplastic-like	S(11): 99.8; S(15): 96.3	24.43	1.65	6.49E-03	1.41	2.54E-02	1.53	8.81E-03
TLDPHIEEQFGSGR	K7UR51	542341	Ribosomal protein s8e family protein	S(12): 100.0	34.6	0.89	5.47E-01	0.54	2.88E-04	0.94	5.93E-01
AAAVAALSSVLTAEQSGSSENLR	K7V5K5	100279855	Uncharacterized protein	S(19): 99.9	75.86	1.44	1.53E-01	1.64	5.41E-03	0.98	8.83E-01
STIGSESASVISSPK	K7W532	100384406	Geminivirus rep-interacting motor	S(9): 96.6; S(13): 96.7	26.81	1.6	1.29E-01	1.22	2.45E-04	1.68	5.35E-06
VAEGNDVSSTGITK	B4FZ13	100275558	Uncharacterized protein	S(8): 100.0; S(9): 100.0	30.11	0.82	6.92E-02	0.68	6.91E-02	0.95	8.38E-01
QEDESDEEDNR	K7TZN7	103653436	Rna-binding protein 25-like isoform	S(5): 100.0	39.93	1.2	1.09E-01	0.99	9.64E-01	1.08	4.05E-01
**Transport**											
GYVVDPELGSNEG	B6SVU3	100281487	ABC transporter C05D10.3 in chromosome III	S(10): 100.0	46.31	0.58	1.02E-03	1.21	2.51E-01	1.04	6.95E-01
DEFSFGNR	K3YR20	101754121	Auxin efflux carrier	S(4): 100.0	31.03	0.77	9.87E-04	0.95	6.83E-01	0.68	1.33E-02
LMSIGSNEAAPTR	K7UJ01	100501607	Pdr-like abc transporter	S(3): 100.0; S(6): 100.0	49.94	0.64	1.52E-02	0.63	7.11E-03	0.83	1.02E-01
SLQSEDALVSQYFQESSSPK	K7VMZ7	100382746	Abc transporter b family member 6-like	S(18): 96.2	21.32	1.15	4.68E-01	1.45	2.91E-03	1.7	4.39E-03
TGFSSGPPPASPPAGGAPSYNSVAPPPDEIQLAK	B4F9M6	100191655	Far upstream element-binding protein	S(11): 99.8	38.37	0.52	4.07E-01				
NAVEDDAESDDEEVPEGR	B4FQI5	100272547	Tpa: vgpw2523 isoform 1	S(9): 100.0	102.26	1.19	3.88E-01	0.61	6.48E-04	0.62	3.63E-03
EIRSPSPTPPPVVGPK	B4FWY2	100281081	Potassium transporter 10	S(4): 100.0; S(6): 100.0	41.67	1.39	1.53E-02	1.08	6.47E-01	1.84	1.68E-03
ADTTLGMSMR	B4FXV2	100281495	Citrate transporter family protein	S(1): 100.0	33.63	1.05	8.74E-01	1.18	3.35E-01	1.73	3.69E-03
ADTTLGMSMR	B4FXV2	100281495	Citrate transporter family protein	T(3): 100.0; S(8): 100.0	33.63	1.05	8.74E-01	1.18	3.35E-01	1.73	3.69E-03
AQELPLKTPENSPK	B4FYD7	100273971	Spore wall protein 2-like isoform	T(8): 100.0; S(12): 100.0	28.78	1.05	6.74E-01	0.68	1.01E-02	0.8	1.95E-02
EPSALSEPSSPK	B4FZY1	100274279	Sodium/hydrogen exchanger	S(3): 100.0; S(6): 100.0; S(10): 99.9	25.17	1.01	9.44E-01	1.51	6.63E-02	0.99	9.67E-01
EPSALSEPSSPK	B4FZY1	100274279	Sodium/hydrogen exchanger	S(3): 100.0; S(10): 99.9	25.17	1.01	9.44E-01	1.51	6.63E-02	0.99	9.67E-01
ATASGGGGGFSGGGGSNMLR	B6SNM4	100280873	Protein transport protein Sec61 beta subunit	S(16): 100.0	20.19	0.77	5.49E-01	0.64	6.95E-02	0.63	6.16E-02
GGGSPVAAVQDASDDGAR	B6U8S7	100285732	Carbohydrate transporter/ sugar porter/ transporter	S(4): 100.0	25.32	1.34	2.63E-02	0.66	2.14E-02	1.11	6.09E-01
FGLASPSSSDEEAK	B6U9U5	100276530	Protein gar2-like	S(9): 96.2	40.07	0.88	4.62E-01	0.67	2.55E-03	0.78	1.11E-02
DQEGGQPTGPEVVADDEVTSHRFTPAR	B8A307	100285151	Citrate transporter family protein	S(20): 92.4; T(24): 92.4	21.48	1.9	6.85E-02	1.68	1.99E-02	1.92	9.69E-03
SVPVGGAFSSK	C0PL03	100285177	Loc100285177 precursor	S(9): 98.8	51		4.66E-01	3.41	2.02E-03		
RVDSLDVESMNVR	C5XBE9	8056191	Potassium transporter	S(4): 100.0	34.76	0.82	3.39E-01	0.69	1.84E-02	1.21	1.15E-01
NSTLPVSNNGSPITEGVSFDDER	E3UJZ2	100505456	Yellow stripe-like transporter	S(11): 100.0	84.2	1.16	4.80E-01	1.23	5.50E-02	0.2	2.11E-04
LGSSAFSLR	F0UXL1	100193700	Sugar transporter protein ERD6-S	S(3): 100.0; S(4): 100.0	38.67	0.95	7.46E-01	1.49	1.33E-04	1.65	7.57E-05
GGAGAGEESGSDHDGVLR	K7URW1	100282396	Solute carrier family facilitated glucose transporter member 8	S(9): 100.0; S(11): 100.0	34.45	1.18	1.52E-01	1.82	1.70E-03	1.62	4.23E-04
GELALVPQSPDR	B4FB91	542392	Ferritin	S(9): 100.0	58.84	0.73	3.12E-02	0.86	1.11E-01	0.39	1.18E-04
AAVEPDQSPITQPGAADASPSR	B4FF32	100193455	Uncharacterized protein	S(8): 100.0; S(19): 98.1	60.32	0.76	7.97E-02	0.65	1.19E-03	0.6	2.18E-03

^a^Sequence of the peptides identified by MS/MS.

^b^Protein Group Accessions in Uniprot database.

^c^Properly phosphoperpetides sites.

^d^Ratio of protein spot abundance in maize SA-treated samples to control samples, based on an average of three replicate gels from independent experiments. A ratio of 1.0 reflects a nonsignificant difference between controls and SA-treated samples (P* *< 0.05). Ratio changes in expression level of at least 1.5-fold.
