# Supplementary material for: Quantitative analysis of changes in the phosphoproteome of maize induced by the plant hormone salicylic acid
Source: Sci Rep. 2015 Dec 11;5:18155. doi: 10.1038/srep18155 (PMC4676064; doi:10.1038/srep18155)
Supplement: Supplementary Information [file srep18155-s1.pdf]

Quantitative analysis of changes in the phosphoproteome of maize induced by the plant hormone salicylic acid

Liuji Wu[1,2†], Xiuli Hu[1†], Shunxi Wang[1,2], Lei Tian[1,2], Yanjie Pang[3], Zanping Han[4], Liancheng Wu[1,2], Yanhui Chen[1,2]*

Figure S1

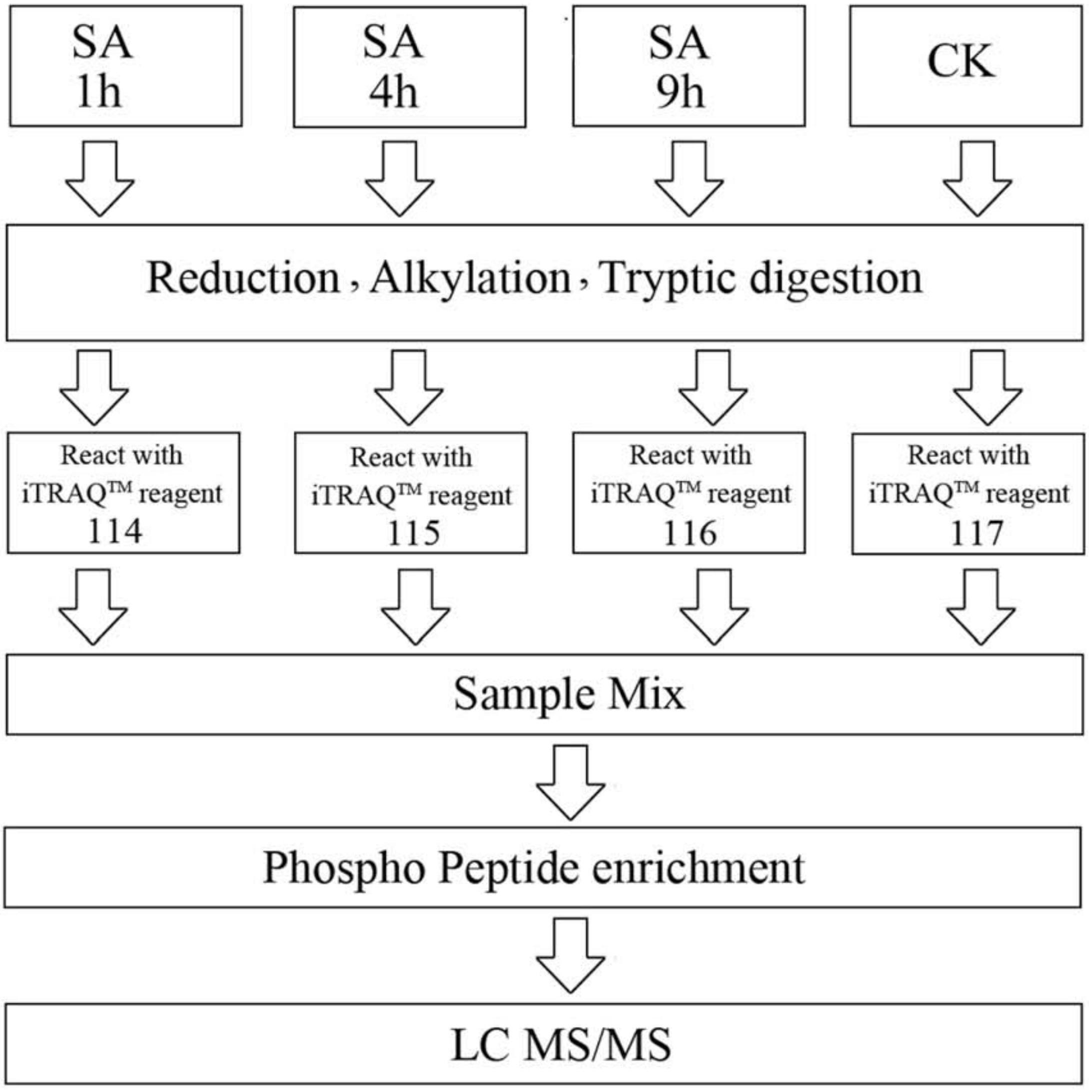

Figure S1: **Experimental strategy employed for the phosphoproteomic analysis, including digestion, labeling, enrichment and analysis**

Figure S2. Volcano plot of the phosphoproteins with phosphorylation levels changes between cases and control.

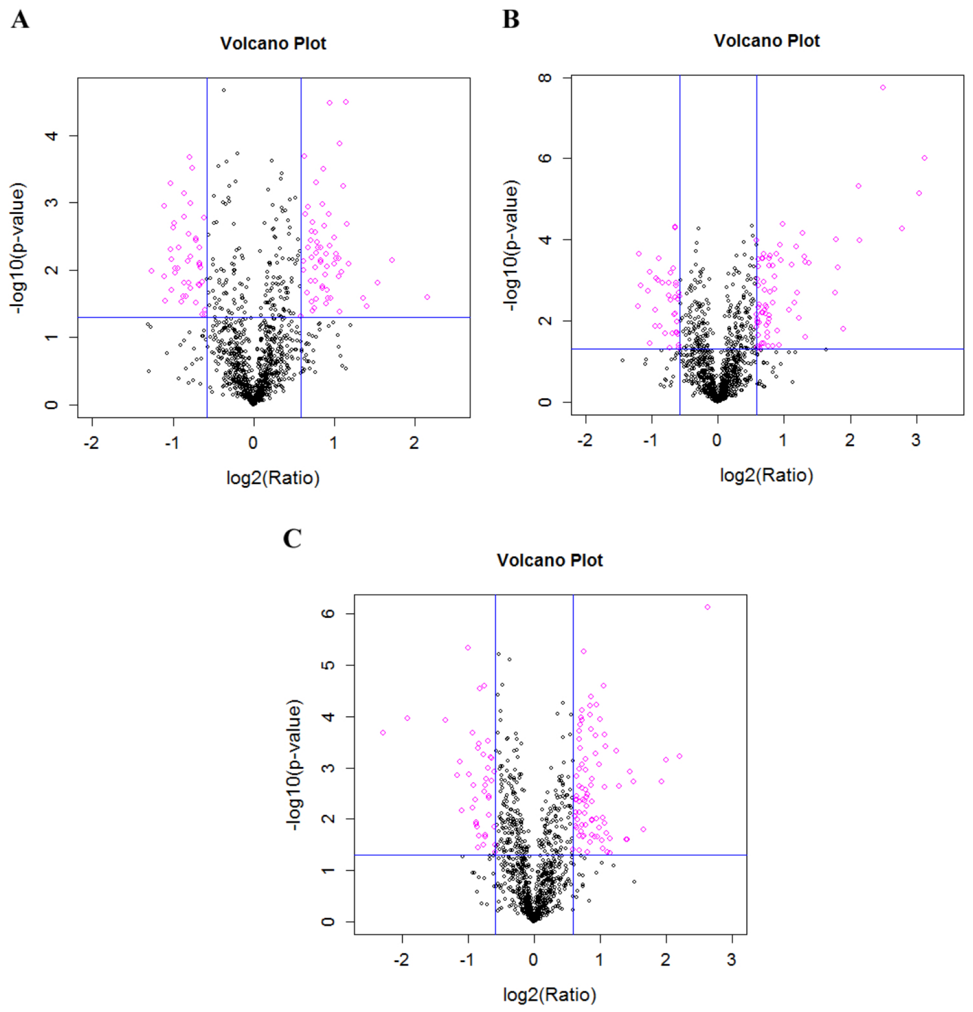

Figure S2: Volcano plot of the phosphoproteins with phosphorylation levels changes between cases and control. A, B and C represent the phosphoproteins with phosphorylation levels changes in 1 h, 4 h and 9 h, respectively. Volcano plot distributions of fold change (log2 [fold change]) (X-axis) and Student's t-test p-values (-log10 [p-value]) (Y-axis). The $-\log_{10}$ (Benjamini–Hochberg corrected *P* value) is plotted against the $\log_2$ (fold change). The non-axial vertical lines denote ±1.5-fold change while the non-axial horizontal line denotes $P = 0.05$, which is our significance threshold (prior to logarithmic transformation).

Table S2. Variance analysis of the phosphopeptides whose levels changed after exposure to SA.

Element	SS	df	MS	F	Sig.
Sample	110.953	1231	0.09	1.383	0.000
Times	0.407	2	0.204	3.124	0.044
Error	157.767	2421	0.065		
Total	4144.138	3655			

$P<0.05$, Level of significance difference; $P<0.01$, markedly significant difference

Table S5. Go distribution of phosphoproteins whose phosphorylation levels were significantly changed after SA treatment

up-regulate

Molecular Function	Numbers	Percent	Biological Process	Numbers	Percent
metal ion binding	44	5.19%	response to stimulus	63	7.43%
nucleotide binding	52	6.13%	metabolic process	113	13.33%
catalytic activity	17	2.00%	regulation of biological process	28	3.30%
enzyme regulator activity	5	0.59%	cell communication	16	1.89%
protein binding	17	2.00%	transport	25	2.95%
DNA binding	6	0.71%	cell organization and biogenesis	12	1.42%
transporter activity	23	2.71%	cellular component movement	3	0.35%
motor activity	3	0.35%	response to stress	9	1.06%
structural molecule activity	3	0.35%	defense response	12	1.42%
unknown	79	9.21%	cell death	2	0.24%
			unknown	71	8.28%

down-regulate

Molecular Function	Numbers	Percent	Biological Process	Numbers	Percent
RNA binding	8	0.94%	transport	26	3.07%
nucleotide binding	44	5.19%	metabolic process	81	9.55%
catalytic activity	54	6.37%	development	13	1.53%
protein binding	14	1.65%	reproduction	7	0.83%
enzyme regulator activity	2	0.24%	response to stimulus	29	3.42%
metal ion binding	19	2.24%	cellular homeostasis	2	0.24%
receptor activity	6	0.71%	regulation of biological process	33	3.89%
signal transducer activity	7	0.83%	cell communication	11	1.30%
transporter activity	5	0.59%	cell proliferation	3	0.35%
structural molecule activity	12	1.42%	development	8	0.94%
DNA binding	3	0.35%	defense response	1	0.12%
unknown	52	6.06%	cell organization and biogenesis	13	1.53%
			cell differentiation	2	0.24%
			unknown	54	6.29%
